# Fine-scale assessment of home ranges and activity patterns for resident black vultures (*Coragyps atratus*) and turkey vultures (*Cathartes aura*)

**DOI:** 10.1371/journal.pone.0179819

**Published:** 2017-07-05

**Authors:** Amanda E. Holland, Michael E. Byrne, A. Lawrence Bryan, Travis L. DeVault, Olin E. Rhodes, James C. Beasley

**Affiliations:** 1 Warnell School of Forestry & Natural Resources, University of Georgia, Athens, Georgia, United States of America; 2 Savannah River Ecology Laboratory, Aiken, South Carolina, United States of America; 3 USDA/APHIS/WS National Wildlife Research Center, Sandusky, Ohio, United States of America; 4 Odum School of Ecology, University of Georgia, Athens, Georgia, United States of America; University of Lleida, SPAIN

## Abstract

Knowledge of black vulture (*Coragyps atratus*) and turkey vulture (*Cathartes aura*) spatial ecology is surprisingly limited despite their vital ecological roles. Fine-scale assessments of space use patterns and resource selection are particularly lacking, although development of tracking technologies has allowed data collection at finer temporal and spatial resolution. Objectives of this study were to conduct the first assessment of monthly home range and core area sizes of resident black and turkey vultures with consideration to sex, as well as elucidate differences in monthly, seasonal, and annual activity patterns based on fine-scale movement data analyses. We collected 2.8-million locations for 9 black and 9 turkey vultures from June 2013 –August 2015 using solar-powered GSM/GPS transmitters. We quantified home ranges and core areas using the dynamic Brownian bridge movement model and evaluated differences as a function of species, sex, and month. Mean monthly home ranges for turkey vultures were ~50% larger than those of black vultures, although mean core area sizes did not differ between species. Turkey vulture home ranges varied little across months, with exception to a notable reduction in space-use in May, which corresponds with timing of chick-rearing activities. Black vulture home ranges and core areas as well as turkey vulture core areas were larger in breeding season months (January–April). Comparison of space use between male and female vultures was only possible for black vultures, and space use was only slightly larger for females during breeding months (February–May). Analysis of activity patterns revealed turkey vultures spend more time in flight and switch motion states (between flight and stationary) more frequently than black vultures across temporal scales. This study reveals substantive variability in space use and activity rates between sympatric black and turkey vultures, providing insights into potential behavioral mechanisms contributing to niche differentiation between these species.

## Introduction

Vultures, as obligate scavengers, provide invaluable ecosystem services by enhancing the flow of nutrients within food webs and reducing transmission of infectious disease through the removal of carrion [1,2,3,4,5,6]. Specializing in carcass consumption [7,8], vultures are adapted to detect and remove carcasses from landscapes more efficiently than any other terrestrial vertebrate scavenger [9]. Because carrion is ephemeral and often randomly distributed [1], vultures have evolved unique adaptations (e.g., broad wings for efficient soaring flight, acute eyesight or enhanced sense of smell) to exploit this spatially and temporally unpredictable resource [10,11]. Unfortunately, vulture populations in many locations have experienced drastic declines over the last few decades [12], making them among the most threatened groups of birds today. Across all vulture species, 73% are exhibiting population declines, 55% are considered either endangered or critically endangered, and 14% are considered near threatened or vulnerable by the International Union for Conservation of Nature [13].

Despite global declines of most other vulture species, populations of black vultures (*Coragyps atratus*) and turkey vultures (*Cathartes aura*) are abundant throughout their distribution [14,15,16], likely due to factors such as the ban of DDT in the United States in the early 1970s, reductions in pesticide use and human persecution, increases in road-killed animals, a greater number of landfills, and a warming climate [17]. Increases in black and turkey vulture populations have led to rises in conflicts between humans and vultures, including the substantial safety risk vultures in flight pose to aircraft due to potential for bird strikes [18,19,20].

Considering their relative ubiquity, importance in ecosystem function, and growing role in human-wildlife conflicts, fine-scale analyses of the spatial ecology of black and turkey vultures are surprisingly limited. Previous home range estimates for black and turkey vultures revealed considerable differences within and among species, as well as among different regions within their respective ranges [21]. While differences in sample size, accuracy of tracking techniques, and estimation methods likely contribute to the substantive differences in home range size reported among studies [22], space use variation also may be due to differences in individual physiology [23], social status [24,25], territoriality [26], or competition [24,25,27,28]. For example, home range sizes of territorial Pyrenean bearded vultures (*Gypaetus barbatus*) were nearly 200 times smaller than those of non-territorial bearded vultures (50 km vs. 10,000 km) [26]. California condor (*Gymnogyps californianus*) home ranges were 5–6 times smaller during breeding season months than in other months presumably because movements were limited and central to nest sites [29]. Conversely, Griffon vulture (*Gyps fulvus*) home ranges were almost three times larger at the end of the breeding season in spring than in winter [30]. In addition, variability in observed space use is likely influenced by seasonal spatio-temporal variation in the distribution and availability of resources [31,32,33,34] as well as in environmental conditions [21], as evidenced by a ~50% reduction in winter versus summer home ranges sizes of black and turkey vultures in the northeastern United States [35].

Knowledge of vulture movement activity patterns (i.e. time spent in flight vs. perched/roosting) is also relatively limited yet can be informative by revealing the amount of search effort required by an individual to obtain important resources within its home range. Outside the migration season, it is reasonable to assume that when a vulture is in flight, it is searching for or returning to some known resource, be it carrion, a nesting location, or roost site. Vultures have shown immense behavioral plasticity in relation to local habitat structure and resource availability [22,30]. For example, black and turkey vultures spent substantially more time in flight, presumably in search of carrion, in heavily forested landscapes than in areas where forage opportunities are more easily detectable, such as agricultural landscapes [32,35]. Additionally, vultures in the southeastern coastal region of the United States spent more time in flight during winter than summer, likely due to the need for increased foraging efforts given reduced ability to detect carrion in colder months via olfactory cues, as well as reduced daylight hours [36].

To date, few studies have quantified home range sizes and activity patterns for resident, non-migratory black and turkey vultures, and none have examined variation in space use at fine temporal scales (e.g., monthly) or between sexes. However, recent advances in global positioning system (GPS) tracking devices and the ease of genetic sex determination for vultures greatly enhance our ability to elucidate factors that underlie variations in movement patterns and space use by black and turkey vultures across an annual cycle. Such data are essential to the further development of our understanding of vulture ecology, benefitting wildlife managers concerned with reducing vulture-related conflicts [33,36,37,38,39] and conservation of these ecologically invaluable species [6].

The objectives of this study, therefore, are to strengthen our understanding of the spatial ecology of black and turkey vultures by comparing home range and core area size estimates between species and sexes at a finer temporal scale (monthly) than previous studies for these species, and to determine the proportion of time spent in flight vs. stationary (roosting, resting, and foraging) to elucidate differences in monthly, seasonal (breeding, summer, and winter), and annual activity patterns. We predict turkey vultures will range across larger areas and spend a greater proportion of time in flight than black vultures, considering the lighter wing loading capacity of turkey vultures, their enhanced sense of smell [40], and higher likelihood of avoiding competitive interactions at carcasses [7]. We further expect turkey vultures will switch between flight and roosting/resting (stationary) states more frequently than black vultures, given that energy expended in flight attempts [41,42] by turkey vultures is less, and also as they typically forage either in pairs or solitarily [7] and are thus more likely to flush from an area when disturbed or challenged at a feeding site. Both black and turkey vultures are monomorphic and both sexes contribute similarly to parental care [7,8]. Thus, we expect space use patterns will not differ as a function of sex, although differences may be evident at finer temporal and spatial scales given possible differences in nutrient requirements for females before and after egg-laying [43]. Additionally, we predict monthly space use for each species will vary over an annual cycle with smaller ranges in winter and breeding season months due to reduced conditions favorable to soaring, and propensity to restrict movements central to nesting locations in breeding seasons [29,44].

## Methods

### Study area

This research was conducted at the Savannah River Site (SRS) located along the border of Georgia and South Carolina in the southeastern United States. The SRS is a 780 km^2^, limited-access, nuclear research facility owned and operated by the U.S. Department of Energy (DOE) [45]. Elevations range from <30 m to 115 m above sea level [45]. Much of the SRS is relatively undisturbed by DOE activities and is forested [45]. The SRS is composed of planted pine forests managed by the U.S. Forest Service, bottomland hardwood, wetland, and various (<5%) industrial areas including five decommissioned nuclear reactors, radioactive materials processing plants, and landfills [45]. The composition of largely undisturbed natural areas makes the SRS an ideal location in which to study resident vulture space use. Black and turkey vultures are abundant on the SRS as it provides important roosting, nesting, and foraging habitat for both species [32,33].

### Vulture trapping & handling

In summer 2013 and spring 2014, we captured 295 vultures using an air-propelled net-cannon at sites baited with wild pig (*Sus scrofa*) or similar carcasses at multiple sites interspersed throughout the SRS. Of these, we selected 27 adult vultures (13 black and 14 turkey) to receive solar-powered 70 g Global System for Mobile Communication/Global Positioning System (GSM/GPS) transmitters (Microwave Telemetry, Columbia, MD) attached via backpack harness. To minimize any effects of transmitter weight on vulture behavior or welfare, only large, adult birds were selected to receive transmitters. In an effort to target resident (i.e. non-migratory) individuals, trapping occurred outside the migration seasons for each species [7,8]. However, after all locations were collected, classification of each individual as either resident vs. migratory was verified by assessing net squared displacement plots (NSD; see below). All captured vultures were affixed with numbered patagial tags for individual identification and handled in compliance with and under approval by the University of Georgia Office of Animal Care and Use Protocol No. A2013 02-004-Y2-A2. For all captured vultures, we collected standard morphological measurements and estimated age class (adult or juvenile) based on coloration and wrinkling of the head [7,8]. Given that black and turkey vultures are sexually monomorphic [7,8], it was not possible to balance ratios of male and female black and turkey vultures from among those randomly selected to receive GPS tracking devices. However, feather and blood samples were collected from captured individuals to aid in sex determination via molecular methods. Sex was determined for transmittered vultures via sex-specific DNA markers amplified by polymerase chain reaction (PCR) according to the methods of Ito et al. [46]. All genetic analyses were conducted at the Savannah River Ecology Laboratory in Aiken, South Carolina.

### Data preparation

Solar-powered GSM/GPS transmitters recorded fixes at variable intervals and reported location (latitude/longitude), speed (knots), course, altitude [meters above sea level (m.a.s.l.)], horizontal and vertical dilution of precision (HDOP, VDOP), and number of satellites used to obtain each fix with a ±23m horizontal error and ±18m vertical error. Our tests of transmitter performance fix collection rates were influenced by a combination of environmental conditions and vulture activity [47]. Fix collection rates increased during days with minimal cloud cover and during times when vultures were flying. Diurnally, fix collection rate was lowest in the evenings and peaked at mid-day [47]. Although fix rates were variable, the fact that fix collection rates were controlled by environmental and behavioral factors in a consistent manner allows us to make comparisons across all individuals. Fixes received during the first two weeks post deployment were excluded from analyses to allow vultures to become accustomed to transmitters and return to normal movement patterns.

For comparisons of monthly space use estimations (i.e. home range and core area) and seasonal and annual activity patterns, data for all vultures were subset and standardized to include only fixes received within equal timeframes. Specifically, for monthly space use and activity patterns comparisons, data from September 1, 2013 to August 31, 2015 were sorted by month based on calendar definitions (i.e., August included all valid fixes collected from August 1 –August 31). For seasonal comparisons of activity patterns, we defined three seasons (breeding, summer and winter; for definitions of these periods see below), and included all fixes received within equal durations with the exception of the first summer. The first summer only included data from the final 53 days of the season because GPS transmitters were deployed in mid-summer. In areas of sympatry, breeding season for black vultures begins, on average, two weeks earlier than turkey vultures (February 1 –June 10 for black vultures and February 15 –June 24 for turkey vultures) [7,8,48]. Therefore, breeding seasons in our analysis included fixes from February 8 –June 9, which represent the median dates of those described for vultures breeding at 32°–33° latitudes [48], and encompassed the range of dates wherein adult black and turkey vultures are both influenced by breeding phenology, and standardized for ease of comparison. Fixes received within the four months preceding and following the breeding season were defined as winter (October 8 –February 7) and summer (June 9 –October 8), respectively. Seasonal data were collected for one and a half summer seasons, two full breeding seasons, and two full winter seasons. For comparisons of annual activity patterns, annual data include locations collected between September 1, 2013 to August 31, 2014 for the first year and September 1, 2014 to August 31, 2015 for the second year.

### Home range and core area estimations

Location data were filtered to remove outliers including fixes with altitudes > 12,000 m, and inconclusive data (e.g., “NegAlt”, “No Fix”, “2D”, “Batt Drain”, and “Low Voltage”),. Speed outliers were determined based on a reasonable assumption that flight speed of a non-migratory vulture would not exceed 90 km/h (25 m/s) [49]. With consideration to altitudinal changes, we used a speed and distance filter [50] to identify and remove locations associated with a minimum required speed >25 m/s. NSD plots were assessed to verify vultures in this analysis were indeed non-migratory individuals. For each vulture, NSD was measured as the straight line distance between the initial roost location and all subsequent roost locations [51]. Specifically, evening roosts were identified by extracting average locations from among stationary fixes received between 20:00:00–04:00:00 hours for each vulture. NSDs were plotted and migration movements visually identified by peaks in NSD within winter months (S1 Fig).

We estimated monthly utilization distributions (UDs) for individual vultures using the dynamic Brownian bridge movement model (dBBMM) [52] with the ‘move’ package [53] in the R program [54]. An advantage of the dBBMM method is that it accounts for both temporal autocorrelation and variation in trajectories between points [52]. To fit the dBBMM to vulture movement paths and allow for comparisons across each model, parameters were standardized (window size = 47, margin = 11, raster = 30) with a mean location error of 23 m based on the manufacturer’s estimate [55]. Static tests revealed vertical and horizontal accuracy (mean = 4.5 m and 7.8 m, respectively) was ~80% and ~60% lower than the manufacturer’s estimate (mean = 22 m and 18 m), respectively [47]; thus, we are confident dBBMMs produced with ±23 m location error delineated reliable boundaries of actual space use.

We delineated monthly core areas and home ranges based on the 50% and 95% UD isopleths, respectively (S2 Fig). Differences in sampling frequencies may introduce bias in UD estimation, and as such, prior to making comparisons, we investigated the relationship between space use and number of fixes. We found no strong correlations between number of fixes and home range (BLVU: R^2^ = 0.02; TUVU: R^2^ = -0.003) or core area (BLVU: R^2^ = -0.007; TUVU: R^2^ = -0.009) estimates indicating no systemic bias existed. Shapiro-Wilk tests revealed that home range and core area sizes were not normally distributed; therefore, we log-transformed the data to reduce the skew of distribution and used unbalanced repeated-measures linear mixed effects models to assess differences in space use across spatial scales (home range and core area). Specifically, at each spatial extent we performed analysis of variance comparisons by (1) species, (2) month, (3) sex, (4) year, (5) month*sex, (7) month*year, and (8) sex*year using the ‘lmerTest’ package [56] in R.

### Activity patterns

We quantified diurnal activity patterns from vulture movement states (in flight vs. stationary) ultimately determined after a series of data refinement procedures. We characterized fixes as day or night based on location-specific estimations of sunrise and sunset using the ‘RAtmosphere’ package [57] in R. Only daytime fixes were included in this analysis. Our field-based accuracy test of the GSM/GPS transmitters revealed the units were reliable indicators of activity state with less than 1% of all locations reported as incorrect movements (i.e., unit reporting speeds >0 knot when stationary or 0 knots when the unit was moving). The GSM/GPS transmitters reported instantaneous speeds in knots (~0.5 m/s). Fixes with instantaneous speeds ≥1 knot were then characterized as “in flight” and all others “stationary”. However, if the movement state of an individual fix was preceded and followed by a series of fixes characterized by the opposite movement state (i.e. a single “in flight” fix between “stationary” fixes), the single fix was removed from analysis as anomalous. Fixes at the end or beginning of a series of “in flight” or “stationary” fixes were re-characterized as “switch” states when preceded or followed by a series of fixes in the opposite state. Proportions of “switch” states were used to compare differences in transitions between flight and stationary behaviors by species. The proportion of locations in transit or stationary states included in subsequent analyses are reported only from the total number of locations unambiguously classified in each state (i.e. excluding “switch” states). Thus, regardless of the number of locations classified as “switch” states, the proportion of “stationary” and “in flight” locations summed to 100% for each individual. Two-sample t-tests were used to determine whether differences in activity rates existed among species across monthly, seasonal, and annual timescales.

## Results

From June 13, 2013 to August 31, 2015, we collected 2,823,627 GPS locations from 26 vultures (13 black and 14 turkey vultures). By the end of the study period, of the 27 vultures that carried GPS units, 13 were still alive; the fates of 11 vultures were unknown as six of the GPS units dropped due to failing attachments and five ceased transmission for unknown reasons; and three vultures were deceased (two by unknown causes and one by gunshot wound). From these data, we estimated 322 monthly 95% home ranges and 50% core areas (S1 Table) for 9 black and 9 turkey vultures (S2 Table) after removing birds with partial data (S3 Table) and months with migratory movements. Two female turkey vultures (TUVU #03 and TUVU #01) exhibited migratory behaviors in at least one winter (S1 Appendix, S3 Fig). Home ranges and core areas estimated for these individuals during their winter migration months were excluded from comparisons with space use by resident vultures in this study. Post-hoc molecular sex determination revealed our sampling included 5 male and 4 female black vultures, and 6 male and 3 female turkey vultures. Given the limited number of female turkey vultures tracked in this study (n = 3), analyses to elucidate differences in space use and activity rates between sexes were only possible for black vultures.

### Monthly 95% home ranges and 50% core area sizes

**Fig 1:**
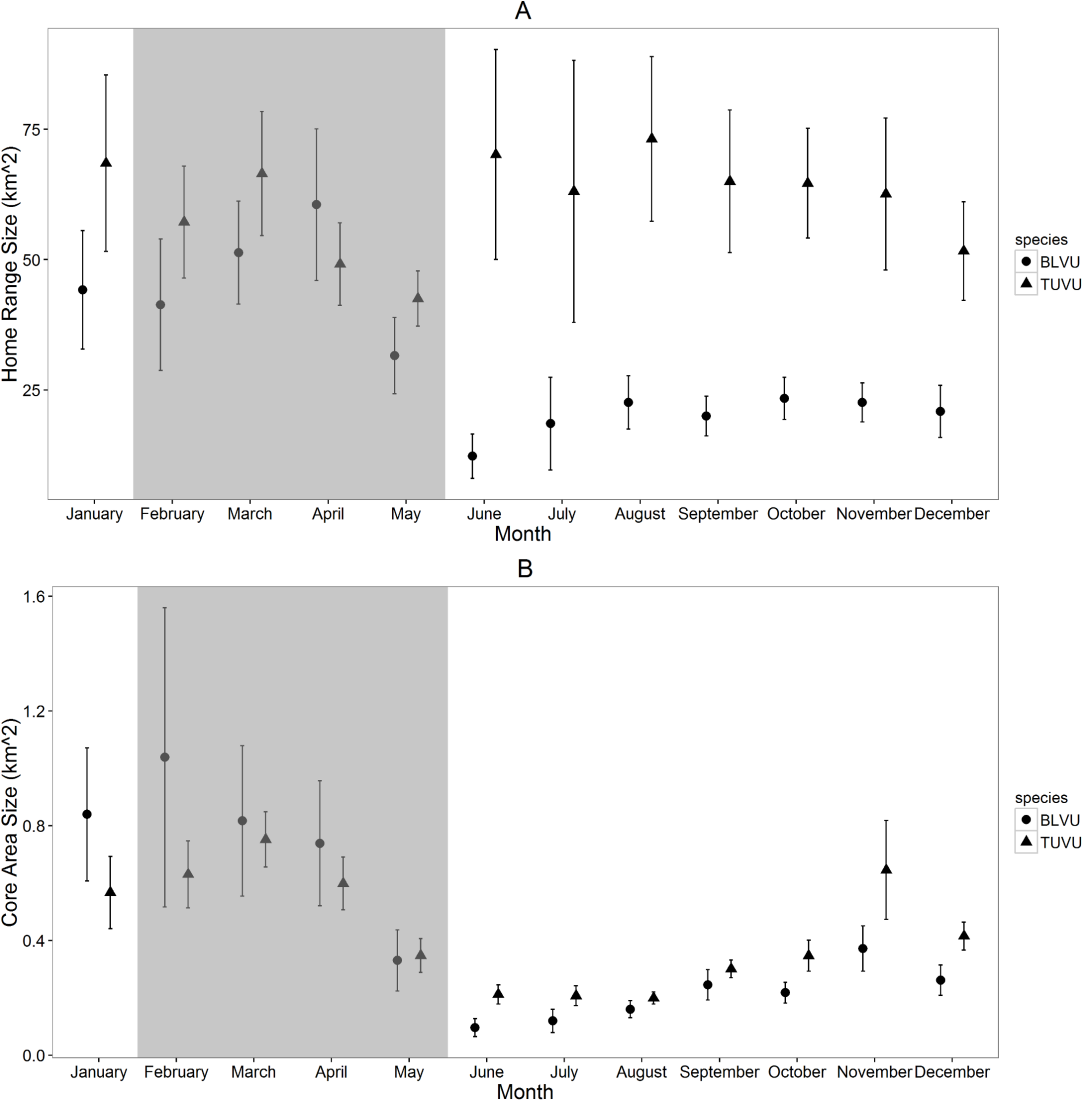
**Mean (± SE) monthly (A) 95% home range and (B) 50% core area sizes (km**^**2**^**) for 9 black vultures (BLVU; total locations = 804,470; mean locations/month = 8,429; range = 895–14,391) and 9 turkey vultures (TUVU; total locations = 1,372,194; mean locations/month = 9,026; range = 531–16,566) calculated from GPS locations collected September 1, 2013 –August 31, 2015.** Shaded region highlights months within the breeding season.

**Fig 2:**
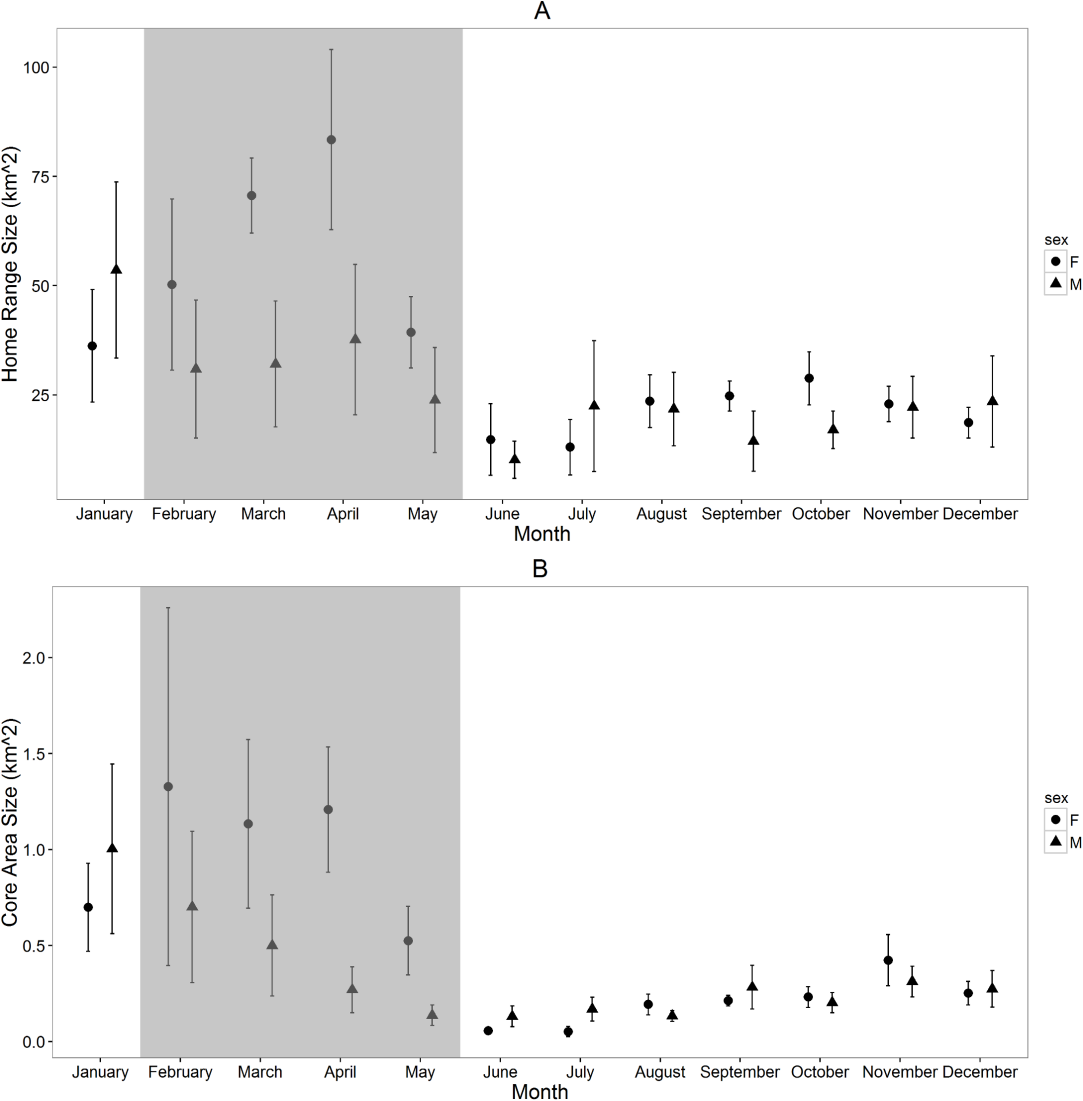
**Mean (± SE) monthly (A) 95% home range and (B) 50% core area sizes (km**^**2**^**) for 5 male (total locations = 437,629; mean locations/month = 8,429; range = 3,164–14,391) and 4 female (total locations = 363,210; mean locations/month = 7,753; range = 895–13,340) resident black vultures (BLVU) calculated from GPS locations collected September 1, 2013 –August 31, 2015.** Shaded region highlights months within the breeding season.

Mean (± SE) monthly home ranges for turkey vultures (61.5 ± 4.3 km^2^) were significantly larger than those of black vultures (30.3 ± 2.6 km^2^; F_1,320_ = 9.5, *P <* 0.01; Fig 1A), although we found no difference between mean monthly core area sizes of turkey vultures (0.42 ± 0.03 km^2^) and black vultures (0.44 ± 0.06 km^2^; F_1,320_ = 2.2, *P =* 0.16; Fig 1B). Monthly space use by black vultures differed significantly over the annual cycle for both home ranges (F_11,150_ = 5.4, *P <* 0.001; Fig 1A) and core areas (F_11,150_ = 5.8, *P <* 0.001; Fig 1B), with home ranges being larger during the months of January through April, and core areas being larger during the months of January through May. For turkey vultures, differences in monthly space use within the annual cycle was evident for core area sizes (F_11,168_ = 7.5, *P <* 0.001; Fig 1B) but not home ranges (F_11,168_ = 0.4, *P =* 0.94; Fig 1A). Turkey vulture core areas were largest in November and January through April, while home ranges were similar across the year except in May when they were substantially smaller and less variable. Finally, differences between male and female black vulture home ranges (F_1,168_ = 0.5, *P =* 0.49; Fig 2A) and core areas (F_1,168_ = 0.1, *P =* 0.71; Fig 2B) were not statistically significant. We also found no evidence for an interactive effect of month and sex for black vulture monthly home ranges (F_1,168_ = 1.2, *P =* 0.29). We did, however, find a significant interactive effect of month and sex for black vulture core areas (F_1,168_ = 3.5, *P <* 0.001). In the middle of the breeding season, during the months of March-May, average monthly home ranges and core areas were notably larger for female black vultures than male black vultures, whereas space-use was similar for both sexes throughout the rest of the annual cycle (Fig 2A and 2B).

### Activity rates

**Fig 3:**
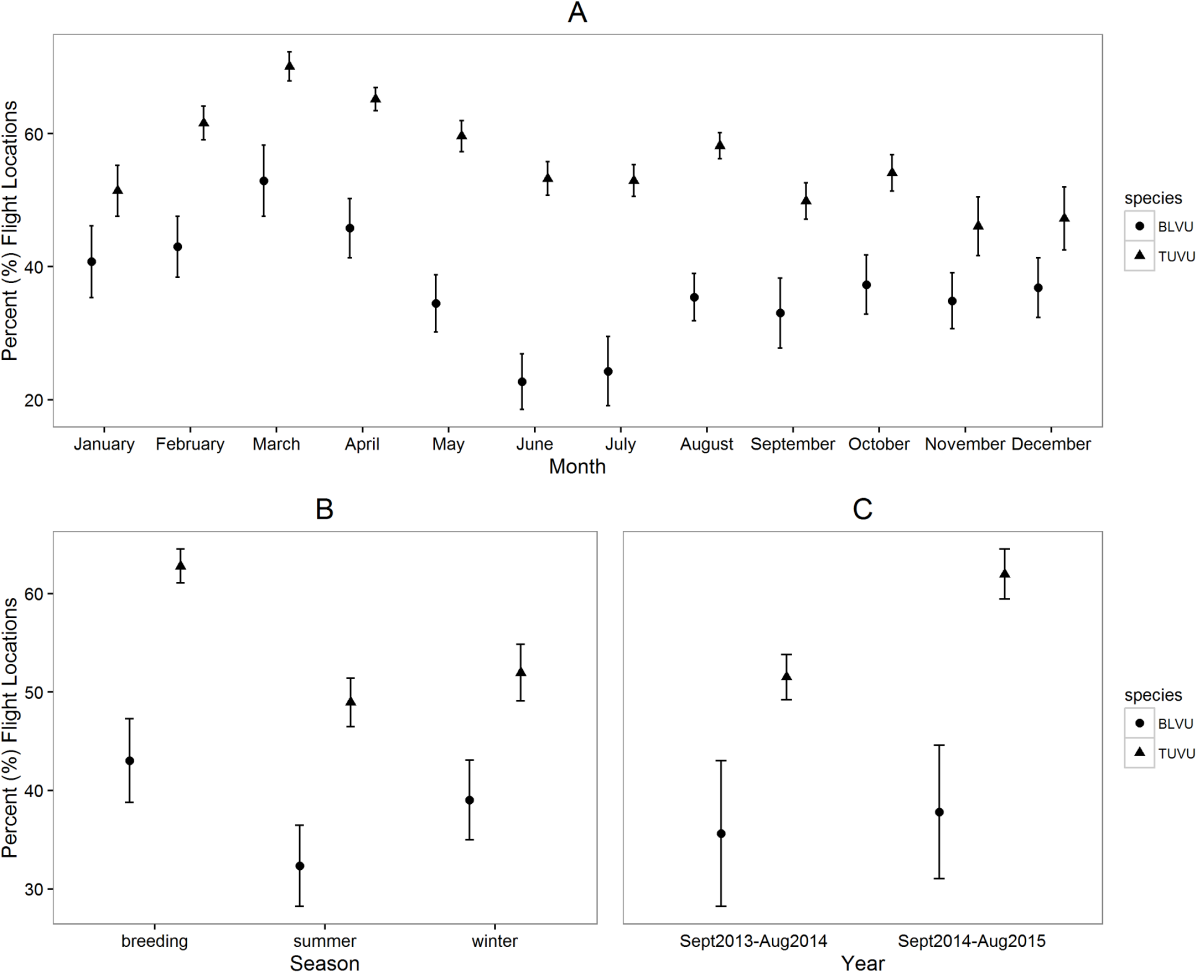
Mean (± 95% CIs) percentage of locations characterized as “in flight” by (A) month, (B) season, and (C) year for 9 black vultures (BLVU; total locations = 990,289; mean = 110,032; range = 29,808–171,932) and 9 turkey vultures (TUVU; total locations = 1,595,225; mean = 177,247; range = 38,694–241,187) calculated from GPS locations collected September 1, 2013 –August 31, 2015.

Our overall comparison of diurnal activity patterns revealed that turkey vultures switched between stationary and flight states (mean ± SE = 7.4 ± 0.3%) ~61% more than black vultures (mean ± SE = 4.5 ± 0.2%; *P <* 0.001; S4 Table). Additionally, turkey vultures spent more time in flight during daylight hours than black vultures across months (F_1,340_ = 12.5, *P <* 0.01; Fig 3A), seasons (F_1,82_ = 11.1, *P <* 0.01; Fig 3B), and years (F_1,25_ = 11.8, *P <* 0.01; Fig 3C). Average (± SE) monthly proportion of time spent in flight during daylight hours was 56 ± 1% for turkey vultures and 37 ± 2% for black vultures (S5 Table). Average (± SE) seasonal proportion of time spent in flight during daylight hours was 54 ± 2% for turkey vultures and 38 ± 2% for black vultures (S6 Table). Average (± SE) annual proportion of time spent in flight during daylight hours was 57 ± 2% for turkey vultures and 39 ± 5% for black vultures (S4 Table).

## Discussion

This study strengthens inferences on the spatial ecology of black and turkey vultures by providing information on the movement behavior of these species at a finer spatio-temporal resolution than any previous study to date. Although both species are obligate scavengers with extensive range overlap, our results reveal substantive differences in home range size and movement behavior exist between these species. These data undoubtedly reflect differences in physiology, behavior, and social structure and thus presumably represent underlying mechanisms of niche differentiation between species.

### Comparisons of home ranges and core area sizes

When compared to estimations of space use in prior studies, or results agree with prior observations that turkey vulture home ranges are generally larger than those of black vultures. However, although we expected turkey vulture space use to be larger than that of black vultures throughout the year, this observation was only true for home ranges and not core areas. Space-use estimations using the Brownian bridge movement model (BBMM) for these species on the southeastern coast of the United States [22] found turkey vulture home ranges and core areas to be six times larger than that observed for black vultures, and an earlier study based on VHF telemetry and using fixed-kernel density (KDE) to quantify home ranges and core areas at the Savannah River Site found mean turkey vulture home ranges to be roughly twice the size of black vulture home ranges [32].

In the southeastern United States, home range and core area sizes of black and turkey vultures show monthly and seasonal variation corresponding with breeding activities. Turkey vulture home range sizes were about twice the size of black vulture home ranges throughout months outside the breeding season; however, during the months of the breeding season, black vulture home range sizes increased significantly, while turkey vulture home ranges reduced slightly, such that home ranges of the two species were relatively similar.

Monthly core area sizes and seasonal variation were similar for these species, being larger in months leading up to and at the height of the breeding season (January–April) than in other months of the year. In our analysis, the breeding season encompassed the months of February through early June (timing of egg-laying to chick fledging [48]). However, breeding behaviors for these species begin as early as January when pairs begin courtship displays and establish nesting territories [48], and we observed substantial changes in home range and core area sizes for both species beginning in January rather than February. Although we were unable to verify breeding status for each vulture in this study, all were adults presumably capable of breeding, and thus potentially influenced by breeding phenology during the months of February through June. It is possible, however, that not all individuals in our study were actually breeding and thus actual monthly differences may have been even stronger among those birds actively breeding and rearing young.

We expected reductions in space use for both species during the breeding seasons with movements central and restricted to nest areas; however, examination of monthly space use revealed movements only were restricted in the months at the end of the breeding season. Turkey vulture and black vulture home ranges were smallest in the months of May and June, respectively, which correspond with timing of chick-rearing [48]. The greatest reduction in space-use for parents might occur during chick-rearing, as demands of newly-hatched and rapidly-growing offspring would require parents to remain near the nest to feed as well as protect and brood during this time when chicks are most vulnerable to predation and exposure. The general demands of breeding appear to affect home range extents differently for these species as turkey vulture space use decreases while black vulture space use increases. Differences in space used by these species are largest when comparing home range extents in the months of the non-breeding season and this is likely owing to the different methods in which these species forage. Because black vultures commonly use communal roosts to enhance their foraging efficiency and follow other vultures to carrion sites [58], site fidelity by black vultures may shift over the annual cycle from communal roosts during the non-breeding season to nest locations during the breeding season.

Core areas of both species were small and less variable during June through October before increasing in both size and variability in the winter months through the breeding season. Furthermore, in the southeastern United States, some vultures migrate northward during the warmer months, likely reducing local abundance and thus decreasing competition for resources. Annual climate variation in the southeastern United States is relatively uniform and observed variations in vulture space use did not appear to correlate with seasonal weather patterns leading us to believe that vulture space use in the southeastern United States is more likely influenced by resource availability and reproduction rather than climate.

Comparisons of space use between sexes were only possible for black vultures in this study due to low numbers of monitored female turkey vultures. Although these differences were not statistically significant, female black vultures generally had larger home ranges and core areas than male black vultures throughout the breeding season (February–May). These months coincide with breeding season and may be associated with an increase in energetic requirements following egg production by females. Space use by male and female black vultures was similar throughout the rest of the year, as would be expected, as energetic requirements for male and female black vultures should be similar for this monomorphic species. These results support our original hypothesis as well as prior understanding of space use by sexually monomorphic species.

### Comparisons of monthly, seasonal, and annual activity patterns

In accordance with our expectation, our results suggest that turkey vultures spend significantly more time in flight than black vultures across all temporal scales measured (monthly, seasonally, and annually). Higher activity patterns in turkey vultures are to be expected considering their lighter wing-loading as the energetic demands of flight are lower for turkey vultures and they are likely to succeed in finding carcasses with random flight searches due to enhanced olfaction as compared to black vultures. In contrast, black vultures spend substantial time in communal roosts for information-exchange in order to enhance their foraging efficiency by following other vultures to carrion sites [58]. We also observed highest activity rates in the breeding season for both black and turkey vultures, which would be expected considering the amount of time both species must spend in courtship flight to establish a mate, as well as maintaining a nest and caring for young.

In conclusion, results of this study show that black and turkey vultures exhibit considerable variability in space use, and that activity rates vary across the annual cycle with space use and activity rates being greatly influenced by demands of the breeding season. Evaluations based on broader-scale sampling regimes may fail to identify important patterns evidencing distinctions. At these finer scales, this study reveals informative details regarding differences in space use and activity patterns by these sympatric species and provides insight into the behavioral, social, and physiological mechanisms underlying niche differentiation between black and turkey vultures.

## Supporting information

S1 Fig Example of net-squared displacement plots from evening roost locations for one migratory turkey vulture (TUVU #03) and one non-migratory turkey vulture (TUVU #06) derived from GPS location data collected from August 16, 2013 to June 9, 2015. Winter seasons (October 7 –February 7) within blue boxes; summer seasons (June 10 –October 6) within yellow boxes; and breeding seasons (February 8 –June 9) within purple boxes. Migratory movements shown by elevated peaks in NSD within winter seasons.
(PDF)

S2 Fig **Example of 95% home ranges and 50% core areas delineated utilizing the dynamic Brownian bridge movement model derived from GPS location data collected from a single black vulture (BLVU #92; blue shades) and a single turkey vulture (TUVU #01; orange shades) in the months of (A) March and (B) August, 2015.** 95% home ranges and 50% core areas are represented in lighter and darker shades, respectively, for the black vulture (blue) and turkey vulture (orange).
(PDF)

S3 Fig Map of vulture movement tracks between evening roost locations for 9 black vultures and 9 turkey vultures derived from GPS location data collected between August 16, 2013 and June 9, 2015. (PDF)

S1 Appendix Details of two migratory turkey vultures tracked in this study. (PDF)

S1 Table Monthly 95% home range sizes (km^2^) and 50% core area sizes (km^2^) derived using the dynamic Brownian Bridge Movement Model (dBBMM) from GPS locations collected September 1, 2013, to August 31, 2015, for 9 black vultures and 9 turkey vultures. (PDF)

S2 Table Birds included in analyses and number of locations received and range of dates over which GPS transmitters were carried for each adult vulture. (PDF)

S3 Table Birds excluded from analyses due to limited (<3 months) or no data collected and number of locations received and range of dates over which GPS transmitters were carried for each adult vulture. (PDF)

S4 Table Proportion (%) of annual diurnal movement states (i.e., transitions between flight and stationary motion states) calculated from GPS locations for 9 black vultures and 9 turkey vultures from September 1, 2013, to August 31, 2015. (PDF)

S5 Table Proportion (%) of monthly diurnal movement states calculated from GPS locations for 9 black vultures and 9 turkey vultures from September 1, 2013, to August 31, 2015. (PDF)

S6 Table Proportion (%) of monthly diurnal movement states calculated from GPS locations for 9 black vultures and 9 turkey vultures from September 1, 2013, to August 31, 2015. (PDF)
