# Supplementary figures and images for: Fine-scale assessment of home ranges and activity patterns for resident black vultures (Coragyps atratus) and turkey vultures (Cathartes aura)
Source: PLoS One. 2017 Jul 5;12(7):e0179819. doi: 10.1371/journal.pone.0179819 (PMC5497974; doi:10.1371/journal.pone.0179819)

# TUVU-03

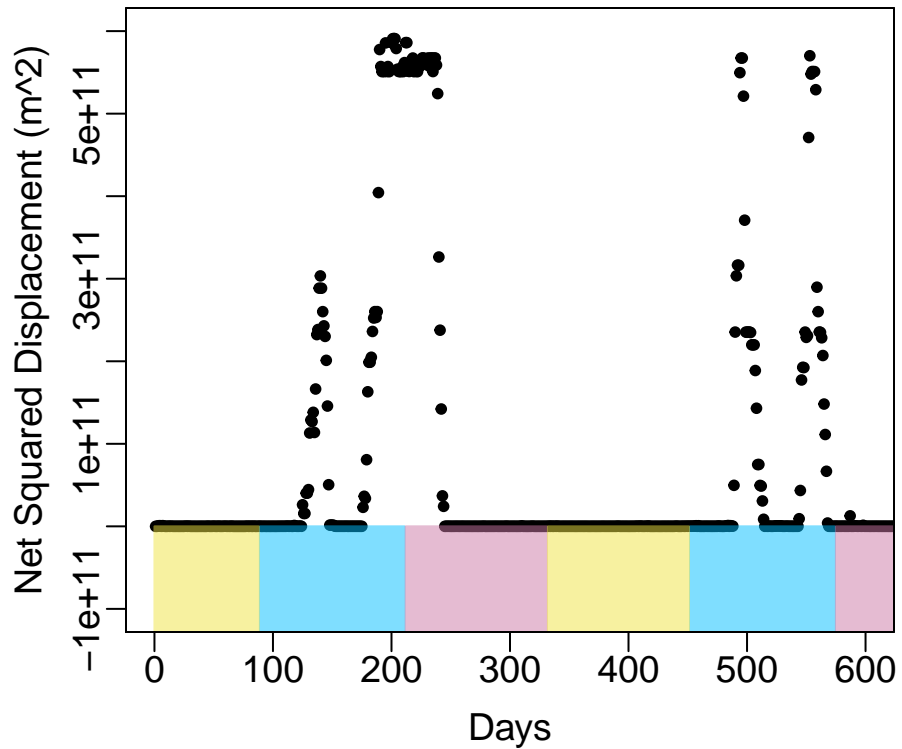

# TUVU-06

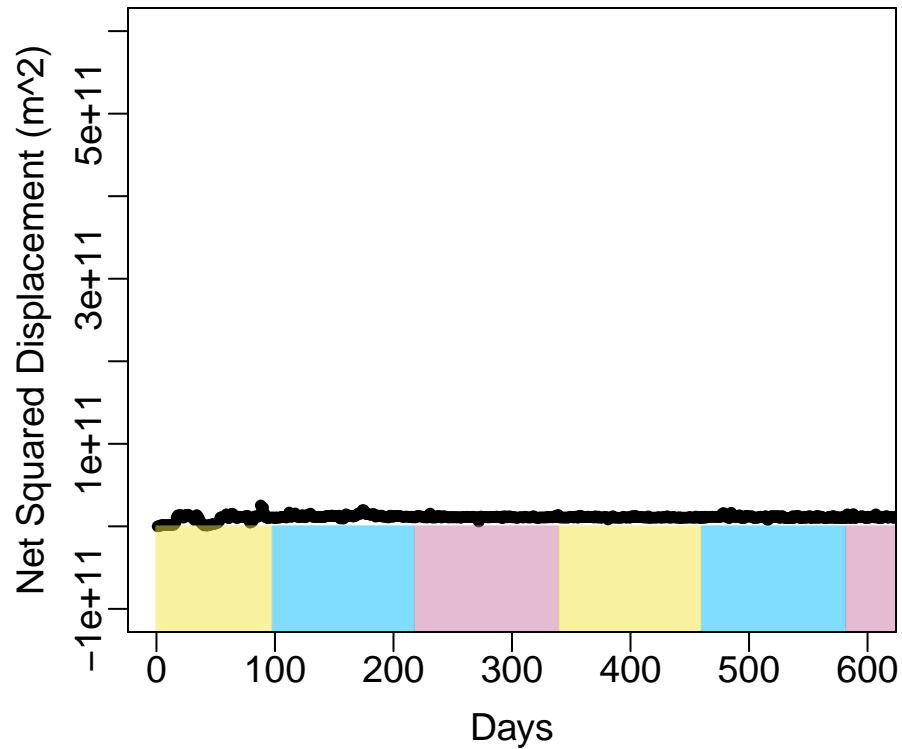

Supplement: S1 Fig — Winter seasons (October 7 –February 7) within blue boxes; summer seasons (June 10 –October 6) within yellow boxes; and breeding seasons (February 8 –June 9) within purple boxes. Migratory movements shown by elevated peaks in NSD within winter seasons. (PDF) [file pone.0179819.s001.pdf]

A

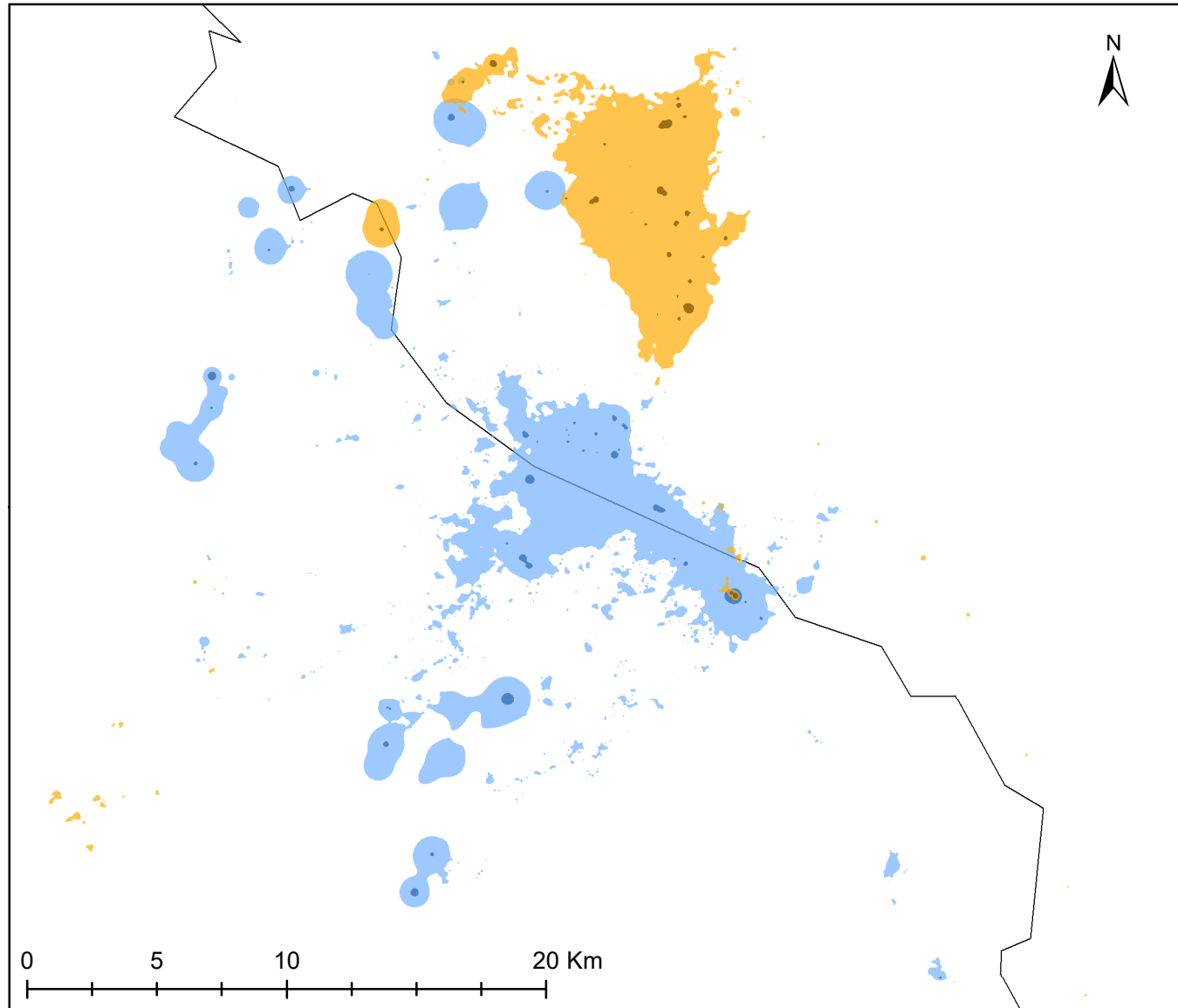

B

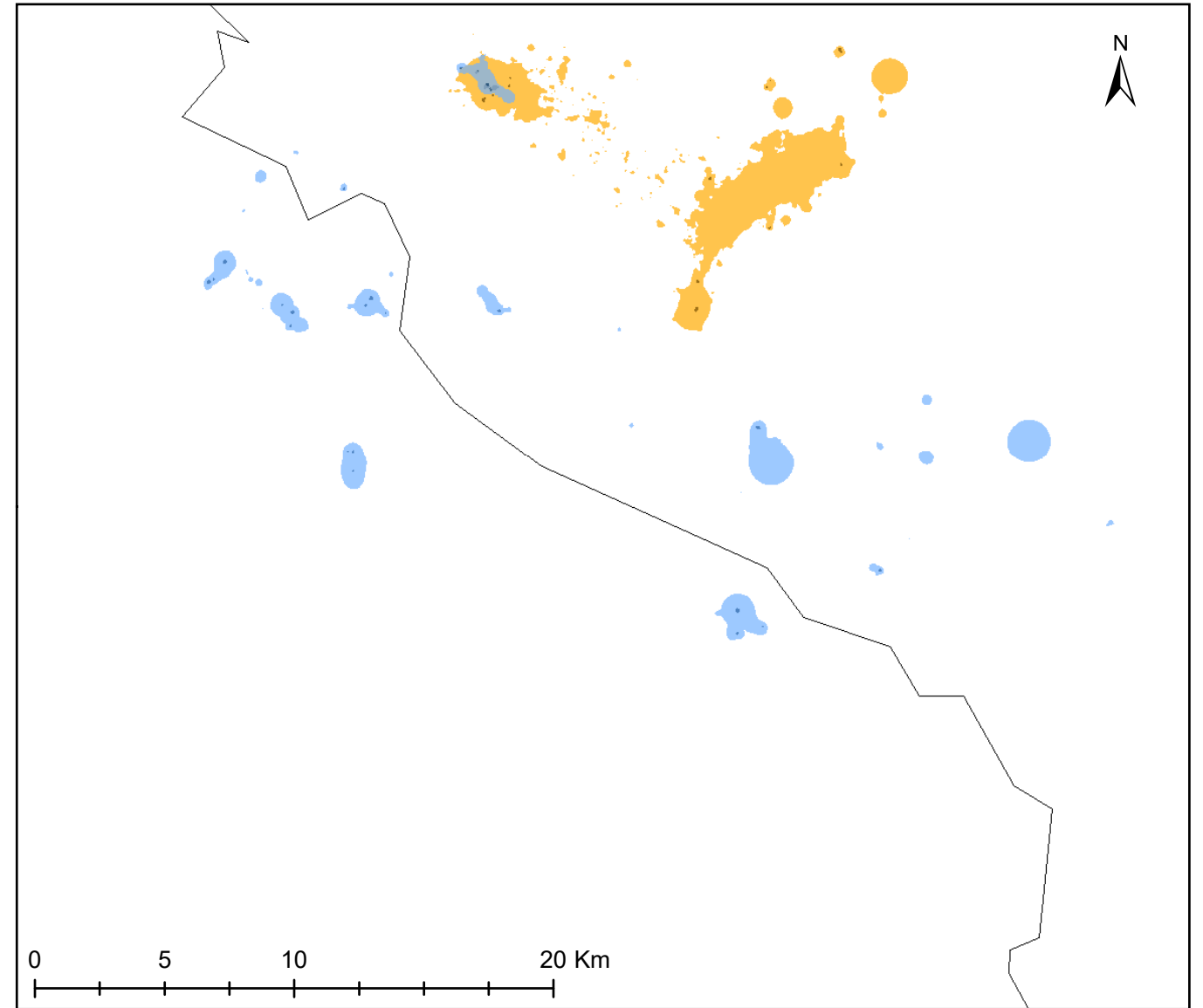

Supplement: S2 Fig — Example of 95% home ranges and 50% core areas delineated utilizing the dynamic Brownian bridge movement model derived from GPS location data collected from a single black vulture (BLVU #92; blue shades) and a single turkey vulture (TUVU #01; orange shades) in the months of (A) March and (B) August, 2015. 95% home ranges and 50% core areas are represented in lighter and darker shades, respectively, for the black vulture (blue) and turkey vulture (orange). (PDF) [file pone.0179819.s002.pdf]

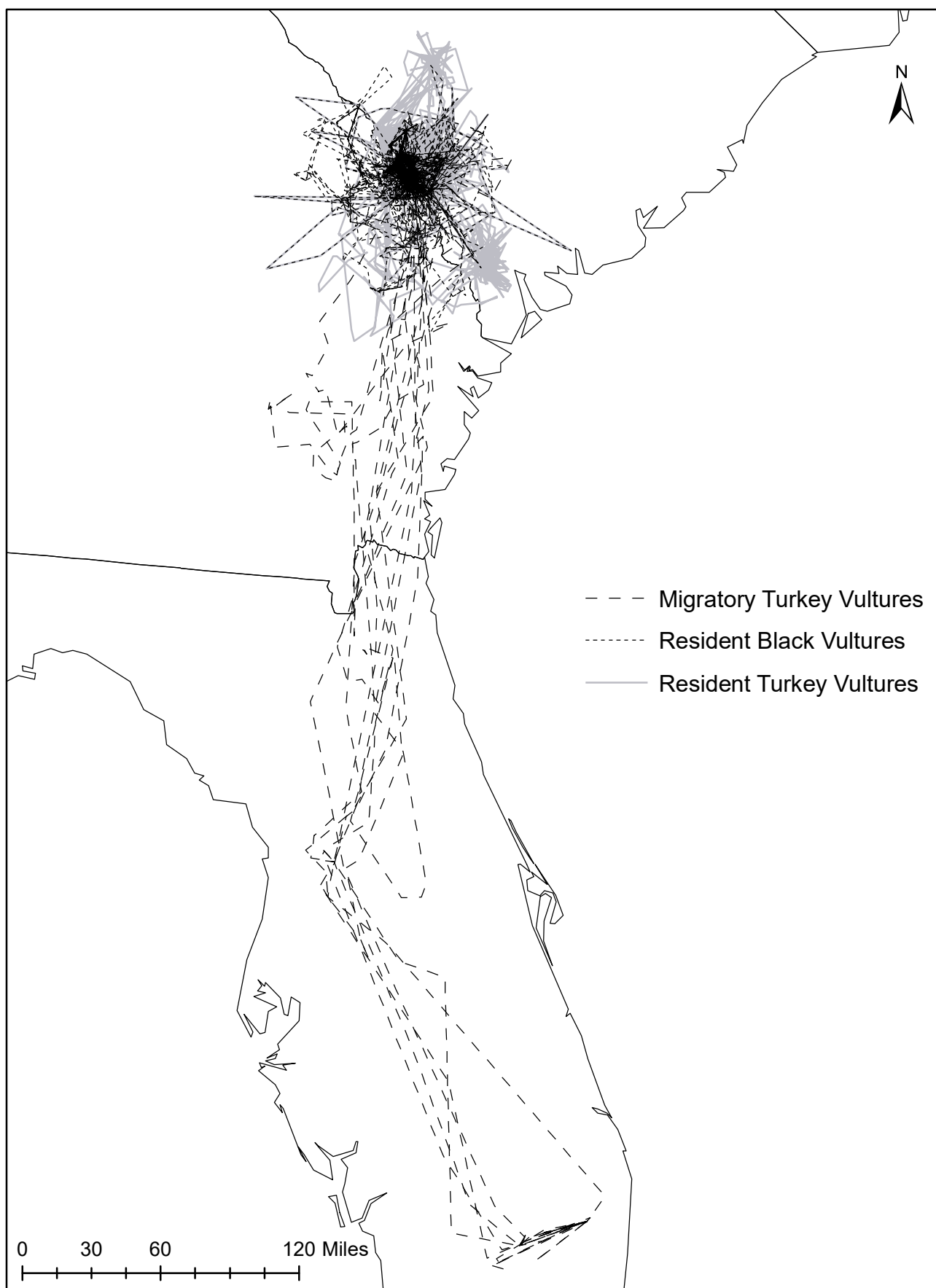

Supplement: S3 Fig — (PDF) [file pone.0179819.s003.pdf]
