# Supplementary material for: Fine-scale assessment of home ranges and activity patterns for resident black vultures (Coragyps atratus) and turkey vultures (Cathartes aura)
Source: PLoS One. 2017 Jul 5;12(7):e0179819. doi: 10.1371/journal.pone.0179819 (PMC5497974; doi:10.1371/journal.pone.0179819)
Supplement: S1 Appendix — (PDF) [file pone.0179819.s004.pdf]

S1 Text. Two turkey vultures showed substantial distances traveled during the winter months, indicative of migratory movements. TUVU #03 migrated south into Florida during both winters, departing November 13, 2013, and returning March 14, 2014, in the first winter, and departing November 15, 2014, and returning February 2, 2015, in the second winter. The farthest distance travelled during migration for this individual was 757 km in year 1 and 774 km in year 2. TUVU #01, migrated 514 km south into Florida during winter 2013, departing October 19, 2013, and returning February 9, 2014, wherein she remained through end of the study (August 31, 2015). Average maximum speed recorded during migration was 50.24 km/hr (min = 43.56 km/hr, max = 56.11 km/hr).
