# Supplementary material for: Fine-scale assessment of home ranges and activity patterns for resident black vultures (Coragyps atratus) and turkey vultures (Cathartes aura)
Source: PLoS One. 2017 Jul 5;12(7):e0179819. doi: 10.1371/journal.pone.0179819 (PMC5497974; doi:10.1371/journal.pone.0179819)
Supplement: S1 Table — (PDF) [file pone.0179819.s005.pdf]

Table S1. Monthly 95% home range sizes (km^2) and 50% core area sizes (km^2) derived using the dynamic Brownian Bridge Movement Model (dBBMM) from GPS locations collected September 1, 2013 to August 31, 2015 for 9 black vultures and 9 turkey vultures. Species: BLVU = Black vulture (*Coragyps atratus*), TUVU = Turkey vulture (*Cathartes aura*); Animal: ID #: patagial tag identification number; Sex: F = female, M = male; Transit: locations wherein the bird was in flight; Stationary: locations wherein the bird was not in flight (i.e., resting, roosting).

Species	Animal	Sex	Year	Month	Home Range	Core Area
BLVU	22	F	1	September	36.38546	0.177499
BLVU	22	F	1	October	39.63946	0.22494
BLVU	22	F	1	November	20.07575	0.216753
BLVU	22	F	1	December	11.28151	0.082044
BLVU	22	F	1	January	6.769757	0.138558
BLVU	22	F	1	February	19.67239	0.078983
BLVU	22	F	1	March	51.85456	0.145702
BLVU	22	F	1	April	178.5538	2.409015
BLVU	22	F	1	May	66.16525	1.287662
BLVU	22	F	1	June	12.16898	0.028804
BLVU	22	F	1	July	15.28004	0.020587
BLVU	22	F	1	August	18.97584	0.040504
BLVU	22	F	2	September	32.49835	0.106994
BLVU	22	F	2	October	59.46741	0.170409
BLVU	22	F	2	November	8.76989	0.072039
BLVU	22	F	2	December	3.56146	0.09979
BLVU	22	F	2	January	21.98809	0.453058
BLVU	22	F	2	February	7.748096	0.088419
BLVU	22	F	2	March	69.72667	0.174884
BLVU	22	F	2	April	93.67302	1.626677
BLVU	22	F	2	May	26.92564	0.086756
BLVU	22	F	2	June	10.31792	0.01777
BLVU	22	F	2	August	5.903933	0.033265
BLVU	47	F	1	September	20.5958	0.229667
BLVU	47	F	1	October	10.60741	0.205003
BLVU	47	F	1	November	14.02269	0.295446
BLVU	47	F	1	December	13.23967	0.219751
BLVU	47	F	1	January	5.382906	0.042919
BLVU	47	F	1	February	59.97674	0.975105
BLVU	47	F	1	March	53.94638	1.102057
BLVU	47	F	1	April	78.53226	0.660206
BLVU	47	F	1	May	9.101051	0.227656
BLVU	47	F	1	June	1.225056	0.041003
BLVU	47	F	1	July	0.392571	0.006442
BLVU	47	F	1	August	48.17359	0.328665

Species	Animal	Sex	Year	Month	Home Range	Core Area
BLVU	47	F	2	September	16.85805	0.257485
BLVU	47	F	2	October	21.79948	0.5189
BLVU	47	F	2	November	31.82894	1.093895
BLVU	47	F	2	December	22.78232	0.235278
BLVU	47	F	2	January	103.0024	1.745332
BLVU	47	F	2	February	158.3756	6.858943
BLVU	47	F	2	March	55.23373	3.049155
BLVU	47	F	2	April	60.97229	1.554458
BLVU	47	F	2	May	52.89327	0.549764
BLVU	47	F	2	June	0.217791	0.015934
BLVU	47	F	2	July	0.150552	0.017329
BLVU	47	F	2	August	21.17588	0.176514
BLVU	57	F	1	September	33.84764	0.191429
BLVU	57	F	1	October	19.73137	0.073509
BLVU	57	F	1	November	16.60181	0.199128
BLVU	57	F	1	December	29.09446	0.236034
BLVU	57	F	1	January	23.70155	0.796537
BLVU	57	F	1	February	21.72004	0.289481
BLVU	92	F	1	September	16.76727	0.338994
BLVU	92	F	1	October	26.7416	0.143074
BLVU	92	F	1	November	36.00723	0.412821
BLVU	92	F	1	December	26.26803	0.569625
BLVU	92	F	1	January	38.54985	1.213339
BLVU	92	F	1	February	24.07427	0.202844
BLVU	92	F	1	March	99.2581	0.820381
BLVU	92	F	1	April	44.70948	0.216749
BLVU	92	F	1	May	44.14533	0.284503
BLVU	92	F	1	June	54.39627	0.125439
BLVU	92	F	1	July	34.63302	0.062563
BLVU	92	F	1	August	31.87924	0.335584
BLVU	92	F	2	September	16.31109	0.190873
BLVU	92	F	2	October	23.64926	0.285571
BLVU	92	F	2	November	33.18185	0.673249
BLVU	92	F	2	December	24.21767	0.316304
BLVU	92	F	2	January	54.08497	0.50307
BLVU	92	F	2	February	60.27262	0.796247
BLVU	92	F	2	March	93.71244	1.507719
BLVU	92	F	2	April	44.01214	0.780311
BLVU	92	F	2	May	36.83247	0.710817
BLVU	92	F	2	June	10.32287	0.101703
BLVU	92	F	2	July	14.6905	0.151372
BLVU	92	F	2	August	15.26503	0.240175

Species	Animal	Sex	Year	Month	Home Range	Core Area
BLVU	08	M	1	September	43.00477	0.736593
BLVU	08	M	1	October	20.50151	0.288333
BLVU	08	M	1	November	47.69274	0.519756
BLVU	08	M	1	December	26.12322	0.26896
BLVU	08	M	1	January	72.43743	2.88176
BLVU	08	M	1	February	62.43805	1.674909
BLVU	08	M	1	March	68.56887	1.467955
BLVU	08	M	1	April	52.56623	0.563132
BLVU	08	M	1	May	75.63944	0.237976
BLVU	12	M	1	September	2.682302	0.057158
BLVU	12	M	1	October	3.502812	0.099979
BLVU	12	M	1	November	11.74762	0.174673
BLVU	12	M	1	December	11.37543	0.190073
BLVU	12	M	1	January	13.25276	0.221293
BLVU	12	M	1	February	3.453405	0.103476
BLVU	12	M	1	March	8.29822	0.107796
BLVU	12	M	1	April	16.36573	0.061737
BLVU	12	M	1	May	4.280589	0.016167
BLVU	12	M	1	June	2.523015	0.051034
BLVU	12	M	1	July	2.5942	0.0933
BLVU	12	M	1	August	1.40625	0.033409
BLVU	12	M	2	September	2.287672	0.077632
BLVU	12	M	2	October	4.07064	0.038738
BLVU	12	M	2	November	4.835139	0.058875
BLVU	12	M	2	December	3.652684	0.029449
BLVU	12	M	2	January	8.17873	0.064686
BLVU	12	M	2	February	10.6904	0.081064
BLVU	12	M	2	March	11.75387	0.054268
BLVU	12	M	2	April	12.68901	0.021585
BLVU	12	M	2	May	5.285268	0.024098
BLVU	12	M	2	June	2.791431	0.0489
BLVU	12	M	2	July	1.926793	0.059556
BLVU	12	M	2	August	2.422116	0.083564
BLVU	48	M	1	September	10.55362	0.230628
BLVU	48	M	1	October	24.68652	0.324831
BLVU	48	M	1	November	39.85386	0.566376
BLVU	48	M	1	December	11.10073	0.069047
BLVU	48	M	1	January	90.42646	1.54193
BLVU	48	M	1	February	5.176836	0.024924
BLVU	48	M	1	March	5.711107	0.044679
BLVU	48	M	1	April	116.5626	0.216541
BLVU	48	M	1	May	11.13895	0.140124

Species	Animal	Sex	Year	Month	Home Range	Core Area
BLVU	48	M	1	June	26.97117	0.168965
BLVU	48	M	1	July	108.8341	0.429634
BLVU	48	M	1	August	55.69478	0.207272
BLVU	108	M	1	June	0.612008	0.032285
BLVU	108	M	1	July	2.305887	0.037795
BLVU	108	M	1	August	8.711761	0.090564
BLVU	108	M	2	September	26.21991	0.502958
BLVU	108	M	2	October	27.36368	0.131529
BLVU	108	M	2	November	12.05835	0.281039
BLVU	108	M	2	December	15.36046	0.446166
BLVU	108	M	2	January	12.20842	0.259547
BLVU	108	M	2	February	8.322862	0.139343
BLVU	108	M	2	March	12.58253	0.148472
BLVU	108	M	2	April	2.985023	0.050932
BLVU	108	M	2	May	3.664274	0.060296
BLVU	108	M	2	June	0.532569	0.027066
BLVU	108	M	2	July	0.171565	0.015456
BLVU	108	M	2	August	5.828951	0.091121
BLVU	126	M	1	June	14.4686	0.160502
BLVU	126	M	1	July	9.693538	0.185108
BLVU	126	M	1	August	40.32172	0.19075
BLVU	126	M	2	September	1.627172	0.091055
BLVU	126	M	2	October	22.05864	0.328827
BLVU	126	M	2	November	17.01224	0.271408
BLVU	126	M	2	December	73.32509	0.638468
BLVU	126	M	2	January	124.8034	1.050313
BLVU	126	M	2	February	95.4043	2.182707
BLVU	126	M	2	March	85.3665	1.177503
BLVU	126	M	2	April	24.91328	0.703392
BLVU	126	M	2	May	42.94266	0.338753
BLVU	126	M	2	June	23.18998	0.426383
BLVU	126	M	2	July	31.64719	0.357706
BLVU	126	M	2	August	37.91073	0.232802
TUVU	01	F	1	September	25.77048	0.212258
TUVU	01	F	1	March	74.81116	0.585442
TUVU	01	F	1	April	47.01644	0.195582
TUVU	01	F	1	May	49.70138	0.160394
TUVU	01	F	1	June	30.34176	0.07596
TUVU	01	F	1	July	31.73342	0.158134
TUVU	01	F	1	August	42.50054	0.178998
TUVU	01	F	2	September	51.03114	0.192739
TUVU	01	F	2	October	60.50856	0.228119

Species	Animal	Sex	Year	Month	Home Range	Core Area
TUVU	01	F	2	November	172.1169	1.535463
TUVU	01	F	2	December	117.3867	0.49061
TUVU	01	F	2	January	131.8589	0.855036
TUVU	01	F	2	February	76.85083	1.246968
TUVU	01	F	2	March	67.47709	0.923601
TUVU	01	F	2	April	52.92709	0.381959
TUVU	01	F	2	May	43.71606	0.36134
TUVU	01	F	2	June	35.54959	0.230878
TUVU	01	F	2	July	25.08115	0.210034
TUVU	01	F	2	August	28.36554	0.210733
TUVU	03	F	1	September	26.70445	0.152437
TUVU	03	F	1	October	27.62839	0.122798
TUVU	03	F	1	April	26.58238	0.414112
TUVU	03	F	1	May	19.8526	0.052693
TUVU	03	F	1	June	22.28511	0.050287
TUVU	03	F	1	July	16.49418	0.061903
TUVU	03	F	1	August	33.7702	0.109677
TUVU	03	F	2	September	43.70766	0.32948
TUVU	03	F	2	October	86.60423	0.214227
TUVU	03	F	2	March	41.25944	0.362249
TUVU	03	F	2	April	35.92697	0.082319
TUVU	03	F	2	May	22.13735	0.131175
TUVU	03	F	2	June	34.32976	0.036883
TUVU	03	F	2	July	20.14984	0.075435
TUVU	03	F	2	August	39.3531	0.110988
TUVU	13	F	1	September	226.885	0.340461
TUVU	13	F	1	October	109.682	0.500816
TUVU	13	F	1	November	116.5755	2.264598
TUVU	06	M	1	September	98.86182	0.36903
TUVU	06	M	1	October	44.02604	0.460327
TUVU	06	M	1	November	28.67069	0.37959
TUVU	06	M	1	December	59.5542	0.554791
TUVU	06	M	1	January	25.55061	0.41606
TUVU	06	M	1	February	25.59812	0.487488
TUVU	06	M	1	March	39.44644	0.243662
TUVU	06	M	1	April	37.45046	0.522472
TUVU	06	M	1	May	35.77338	0.134329
TUVU	06	M	1	June	65.8275	0.165524
TUVU	06	M	1	July	32.56598	0.113785
TUVU	06	M	1	August	34.71302	0.132523
TUVU	06	M	2	September	19.92862	0.177493
TUVU	06	M	2	October	33.22145	0.21972

Species	Animal	Sex	Year	Month	Home Range	Core Area
TUVU	06	M	2	November	29.23456	0.588298
TUVU	06	M	2	December	30.49198	0.26085
TUVU	06	M	2	January	39.70316	0.564474
TUVU	06	M	2	February	34.70924	0.567418
TUVU	06	M	2	March	31.06979	1.604646
TUVU	06	M	2	April	27.15696	0.660463
TUVU	06	M	2	May	38.08018	0.801534
TUVU	06	M	2	June	28.24499	0.162512
TUVU	06	M	2	July	34.67658	0.261145
TUVU	06	M	2	August	31.71504	0.17537
TUVU	60	M	1	September	111.0426	0.375933
TUVU	60	M	1	October	73.52571	0.517216
TUVU	60	M	1	November	142.4141	0.963351
TUVU	60	M	1	December	54.6332	0.561876
TUVU	60	M	1	January	53.77608	0.295667
TUVU	60	M	1	February	106.5911	0.531993
TUVU	60	M	1	March	115.6291	0.778559
TUVU	60	M	1	April	144.7547	0.698524
TUVU	60	M	1	May	72.44932	0.285706
TUVU	60	M	1	June	45.89535	0.218318
TUVU	60	M	1	July	23.05887	0.04916
TUVU	60	M	1	August	92.87206	0.103705
TUVU	60	M	2	September	56.36045	0.278325
TUVU	60	M	2	October	138.3106	0.507281
TUVU	60	M	2	November	36.67388	0.272871
TUVU	60	M	2	December	52.73412	0.352605
TUVU	60	M	2	January	156.3954	1.134754
TUVU	60	M	2	February	127.8075	0.590275
TUVU	60	M	2	March	141.925	1.053154
TUVU	60	M	2	April	90.94666	1.461631
TUVU	60	M	2	June	54.91728	0.11733
TUVU	60	M	2	July	83.98897	0.097714
TUVU	60	M	2	August	76.24484	0.133871
TUVU	75	M	1	September	119.7884	0.121423
TUVU	75	M	1	October	134.1087	0.939767
TUVU	75	M	1	November	48.91576	0.080529
TUVU	75	M	1	December	11.35742	0.146515
TUVU	75	M	1	January	16.53487	0.282066
TUVU	75	M	1	February	24.67161	0.311267
TUVU	75	M	1	March	35.14899	0.660198
TUVU	75	M	1	April	38.84341	0.383078
TUVU	75	M	1	May	39.39505	0.223841

Species	Animal	Sex	Year	Month	Home Range	Core Area
TUVU	75	M	1	June	77.69896	0.197388
TUVU	75	M	1	July	88.97249	0.262673
TUVU	75	M	1	August	154.7801	0.208027
TUVU	75	M	2	September	63.20231	0.459832
TUVU	75	M	2	October	91.82743	0.241498
TUVU	75	M	2	November	92.67748	0.596618
TUVU	75	M	2	December	93.67966	0.733126
TUVU	75	M	2	January	123.0943	1.189901
TUVU	75	M	2	February	89.63531	1.5813
TUVU	75	M	2	March	42.43233	0.79854
TUVU	75	M	2	April	33.0853	0.436948
TUVU	75	M	2	May	27.13963	0.350873
TUVU	75	M	2	June	12.51306	0.17567
TUVU	75	M	2	July	10.46635	0.220089
TUVU	90	M	1	September	17.51711	0.31538
TUVU	90	M	1	October	19.7691	0.223897
TUVU	90	M	1	November	61.41529	0.627764
TUVU	90	M	1	December	55.13003	0.529918
TUVU	90	M	1	January	166.6859	1.283693
TUVU	90	M	1	February	57.86101	0.723678
TUVU	90	M	1	March	42.55975	0.932745
TUVU	90	M	1	April	33.30223	0.795406
TUVU	90	M	1	May	19.83962	0.491546
TUVU	90	M	1	June	33.96339	0.275855
TUVU	90	M	1	July	25.55496	0.275816
TUVU	90	M	1	August	24.67824	0.26696
TUVU	90	M	2	September	39.97183	0.457712
TUVU	90	M	2	October	31.42443	0.225067
TUVU	90	M	2	November	45.66754	0.429017
TUVU	90	M	2	December	74.09961	0.474384
TUVU	90	M	2	January	44.76073	0.060788
TUVU	90	M	2	February	26.21429	0.588718
TUVU	90	M	2	March	13.4671	1.002822
TUVU	90	M	2	April	32.02017	1.143186
TUVU	90	M	2	May	33.05929	0.71706
TUVU	90	M	2	June	76.86479	0.471798
TUVU	90	M	2	July	69.38406	0.638618
TUVU	90	M	2	August	32.62004	0.346035
TUVU	91	M	1	September	79.14929	0.232395
TUVU	91	M	1	October	51.67277	0.345201
TUVU	91	M	1	November	9.15241	0.157092
TUVU	91	M	1	December	20.98445	0.253463

Species	Animal	Sex	Year	Month	Home Range	Core Area
TUVU	91	M	1	January	18.89256	0.298797
TUVU	91	M	1	February	64.0706	0.400677
TUVU	91	M	1	March	128.276	0.279145
TUVU	91	M	1	April	45.31024	0.428593
TUVU	91	M	1	May	41.93361	0.521527
TUVU	91	M	1	June	30.06633	0.26855
TUVU	91	M	1	July	13.57596	0.183863
TUVU	91	M	1	August	124.1488	0.172993
TUVU	91	M	2	September	54.71795	0.560874
TUVU	91	M	2	October	62.47028	0.275761
TUVU	91	M	2	November	25.55697	0.254323
TUVU	91	M	2	December	43.5501	0.344376
TUVU	91	M	2	January	38.41398	0.087177
TUVU	91	M	2	February	46.21099	0.11905
TUVU	91	M	2	March	133.2863	0.792909
TUVU	91	M	2	April	48.39094	0.840457
TUVU	91	M	2	May	72.84576	0.286863
TUVU	91	M	2	June	130.1736	0.284399
TUVU	91	M	2	July	101.7474	0.209188
TUVU	91	M	2	August	197.266	0.332063
TUVU	123	M	1	June	349.115	0.518128
TUVU	123	M	1	July	422.7896	0.240914
TUVU	123	M	1	August	174.9421	0.204021
TUVU	123	M	2	September	5.394169	0.235554
TUVU	123	M	2	October	5.032932	0.175854
TUVU	123	M	2	November	4.615338	0.244797
TUVU	123	M	2	December	6.060012	0.277709
TUVU	123	M	2	January	6.17557	0.334084
TUVU	123	M	2	February	6.096075	0.415719
TUVU	123	M	2	March	24.27655	0.510108
TUVU	123	M	2	April	43.47567	0.530802
TUVU	123	M	2	May	79.36845	0.350334
TUVU	123	M	2	June	94.43807	0.144296
TUVU	123	M	2	July	9.122276	0.252231
TUVU	123	M	2	August	9.112253	0.301805
