# Supplementary material for: Fine-scale assessment of home ranges and activity patterns for resident black vultures (Coragyps atratus) and turkey vultures (Cathartes aura)
Source: PLoS One. 2017 Jul 5;12(7):e0179819. doi: 10.1371/journal.pone.0179819 (PMC5497974; doi:10.1371/journal.pone.0179819)
Supplement: S2 Table — (PDF) [file pone.0179819.s006.pdf]

Table S2. Birds included in analyses and number of locations received and range of dates over which GPS transmitters were carried for each adult vulture. Species: BLVU = Black Vulture (*Coragyps atratus*), TUVU = Turkey Vulture (*Cathartes aura*); ID: patagial tag identification number; Sex: F = female, M = male. Transmitter deployment date: month/day/year.

**Species**	**ID**	**Sex**	**No. Locations**	**Transmitter Deployment Start Date**	**End Date**	**Status**
BLVU	8	M	102,537	6/18/2013	6/18/2014	Transmission ceased; bird fate unknown.
BLVU	12	M	178,571	6/20/2013	9/1/2015	Active at end of study.
BLVU	48	M	96,043	7/3/2013	9/22/2014	Transmitter dropped; bird fate unknown; transmitter redeployed.
BLVU	108	M	91,654	4/21/2014	9/1/2015	Active at end of study.
BLVU	126	M	128,921	5/1/2014	9/1/2015	Active at end of study.
BLVU	22	F	121,498	6/20/2013	9/1/2015	Deceased.
BLVU	47	F	121,046	7/3/2013	9/1/2015	Active at end of study.
BLVU	57	F	31,931	7/5/2013	3/21/2014	Deceased; transmitter redeployed.
BLVU	92	F	181,466	8/2/2013	9/1/2015	Active at end of study.
TUVU	6	M	215,442	6/17/2013	8/27/2015	Active at end of study.
TUVU	60	M	225,904	7/5/2013	9/1/2015	Active at end of study.

Table S1 (continued). Birds included in analyses and number of locations received and range of dates over which GPS transmitters were carried for each adult vulture. Species: BLVU = Black Vulture (*Coragyps atratus*), TUVU = Turkey Vulture (*Cathartes aura*); ID: patagial tag identification number; Sex: F = female, M = male. Transmitter deployment date: year/month/day.

**Species**	**ID**	**Sex**	**No. Locations**	**Transmitter Deployment**		**Status**
				**Start Date**	**End Date**	
TUVU	75	M	201,902	7/9/2013	9/1/2015	Active at end of study.
TUVU	90	M	185,527	7/30/2013	9/1/2015	Active at end of study.
TUVU	91	M	202,737	7/31/2013	9/1/2015	Active at end of study.
TUVU	123	M	147,388	4/23/2014	9/1/2015	Active at end of study.
TUVU	1	F	257,753	6/26/2013	9/1/2015	Active at end of study.
TUVU	3	F	258,859	6/26/2013	9/1/2015	Active at end of study.
TUVU	13	F	41,625	6/27/2013	12/21/2013	Transmission ceased; bird fate unknown.
