# Supplementary material for: Fine-scale assessment of home ranges and activity patterns for resident black vultures (Coragyps atratus) and turkey vultures (Cathartes aura)
Source: PLoS One. 2017 Jul 5;12(7):e0179819. doi: 10.1371/journal.pone.0179819 (PMC5497974; doi:10.1371/journal.pone.0179819)
Supplement: S3 Table — (PDF) [file pone.0179819.s007.pdf]

Table S3. Birds excluded from analyses due to limited (<3 months) or no data collected and number of locations received and range of dates over which GPS transmitters were carried for each adult vulture (sex undetermined). Species: BLVU = Black Vulture (*Coragyps atratus*), TUVU = Turkey Vulture (*Cathartes aura*); ID: patagial tag identification number; transmitter deployment date: month/day/year.

**Species**	**ID**	**No. Locations**	**Transmitter Deployment**		**Status**
			**Start Date**	**End Date**	
BLVU	4	0	6/17/2013	6/17/2013	Transmission ceased; bird fate unknown.
BLVU	44	8,489	7/3/2013	8/26/2013	Transmitter dropped; bird fate unknown; transmitter redeployed.
BLVU	46	20,560	7/3/2013	9/21/2013	Transmitter dropped; bird fate unknown; transmitter redeployed.
BLVU	65	2,833	7/8/2013	7/25/2013	Deceased; transmitter redeployed.
TUVU	0	225	6/13/2013	7/10/2013	Transmission ceased; bird fate unknown.
TUVU	2	143	6/17/2013	6/17/2013	Transmission ceased; bird fate unknown.
TUVU	10	280	6/18/2013	6/19/2013	Transmitter dropped; bird fate unknown; transmitter redeployed.

Table S2 (continued). Birds excluded from analyses due to limited (<3 months) or no data collected and number of locations received and range of dates over which GPS transmitters were carried for each adult vulture (sex undetermined). Species: BLVU = Black Vulture (*Coragyps atratus*), TUVU = Turkey Vulture (*Cathartes aura*); ID: patagial tag identification number; transmitter deployment date: month/day/year.

**Species**	**ID**	**No. Locations**	**Transmitter Deployment**		**Status**
			**Start Date**	**End Date**	
TUVU	19	268	6/27/2013	6/27/2013	Transmitter dropped; bird fate unknown; transmitter redeployed.
TUVU	76	25	7/9/2013	7/10/2013	Transmitter dropped; bird fate unknown; transmitter redeployed.
