# Supplementary material for: Fine-scale assessment of home ranges and activity patterns for resident black vultures (Coragyps atratus) and turkey vultures (Cathartes aura)
Source: PLoS One. 2017 Jul 5;12(7):e0179819. doi: 10.1371/journal.pone.0179819 (PMC5497974; doi:10.1371/journal.pone.0179819)
Supplement: S4 Table — (PDF) [file pone.0179819.s008.pdf]

Table S4. Proportion (%) of annual diurnal movement states calculated from GPS locations for 9 black vultures and 9 turkey vultures from September 2013 – August 2015. For transit and stationary values, white cell values report diurnal activity rates for two years (i.e., pooled data over year 1 and year 2), grey cell values report diurnal activity rates for a single year (i.e., no data were collected within either year 1 or year 2), black cells indicate no data were collected over a full year. Proportion (%) of switch states (i.e., transitions between flight and stationary motion states) calculated from all available GPS locations. Species: BLVU = Black Vulture (*Coragyps atratus*), TUVU = Turkey Vulture (*Cathartes aura*); ID #: patagial tag identification number; Sex: F = female, M = male; Transit: locations wherein the bird was in flight; Stationary: locations wherein the bird was not in flight (i.e., resting, roosting).

Species	ID #	Sex	Transit	Stationary	Switch States (%)
BLVU	22	F	46.66	53.34	5.91
BLVU	47	F	41.38	58.62	5.10
BLVU	57	F	31.08	68.92	3.96
BLVU	92	F	44.08	55.92	4.20
BLVU	08	M	66.43	33.57	4.37
BLVU	12	M	22.13	77.87	4.40
BLVU	48	M	28.50	71.50	4.63
BLVU	108	M	20.09	79.91	3.34
BLVU	126	M	48.74	51.26	4.24
TUVU	01	F	60.92	39.08	7.86
TUVU	03	F	64.66	35.34	6.30
TUVU	13	F			5.89
TUVU	06	M	51.49	48.51	7.77
TUVU	60	M	57.22	42.78	6.66
TUVU	75	M	61.55	38.45	7.67
TUVU	90	M	58.82	41.18	8.20
TUVU	91	M	52.72	47.28	7.37
TUVU	123	M	50.06	49.94	8.45
