# Supplementary material for: Fine-scale assessment of home ranges and activity patterns for resident black vultures (Coragyps atratus) and turkey vultures (Cathartes aura)
Source: PLoS One. 2017 Jul 5;12(7):e0179819. doi: 10.1371/journal.pone.0179819 (PMC5497974; doi:10.1371/journal.pone.0179819)
Supplement: S5 Table — (PDF) [file pone.0179819.s009.pdf]

Table S5. Proportion (%) of monthly diurnal movement states calculated from GPS locations for 9 black vultures and 9 turkey vultures from September 2013 – August 2015. White cell values report activity rates within that month over two years (i.e., pooled data over year 1 and year 2). Grey cell values report activity rates within that month for a single year (i.e., no data were collected within that month in either year 1 or year 2). Black cells are months within which no data were collected. Species: BLVU = Black Vulture (*Coragyps atratus*), TUVU = Turkey Vulture (*Cathartes aura*); ID #: patagial tag identification number; Sex: F = female, M = male; Transit: locations wherein the bird was in flight; Stationary: locations wherein the bird was not in flight (i.e., resting, roosting).

			January		February		March		April		May		June		July		August		September		October		November		December	
Species	ID #	Sex	Transit	Stationary	Transit	Stationary	Transit	Stationary	Transit	Stationary	Transit	Stationary	Transit	Stationary	Transit	Stationary	Transit	Stationary	Transit	Stationary	Transit	Stationary	Transit	Stationary	Transit	Stationary
BLVU	22	F	37.16	62.84	41.99	58.01	45.28	54.72	45.13	54.87	39.54	60.46	44.52	55.48	50.86	49.14	50.41	49.59	47.37	52.63	54.50	45.50	42.23	57.77	39.13	60.87
BLVU	47	F	62.70	37.30	64.16	35.84	64.79	35.21	59.18	40.82	39.53	60.47	6.06	93.94	0.63	99.37	44.36	55.64	23.29	76.71	26.95	73.05	34.66	65.34	53.40	46.60
BLVU	57	F	22.12	77.88	35.38	64.62													31.76	68.24	28.57	71.43	39.21	60.79	24.27	75.73
BLVU	92	F	41.06	58.94	46.60	53.40	67.21	32.79	49.23	50.77	41.43	58.57	26.21	73.79	28.64	71.36	43.32	56.68	50.42	49.58	50.18	49.82	44.83	55.17	32.04	67.96
BLVU	08	M	74.25	25.75	61.69	38.31	68.76	31.24	74.22	25.78	67.82	32.18							68.70	31.30	57.97	42.03	63.90	36.10	70.16	29.84
BLVU	12	M	32.02	67.98	29.13	70.87	39.18	60.82	32.01	67.99	26.74	73.26	16.03	83.97	11.61	88.39	13.32	86.68	12.89	87.11	16.45	83.55	16.16	83.84	25.42	74.58
BLVU	48	M	41.11	58.89	28.31	71.69	24.73	75.27	30.58	69.42	19.58	80.42	27.48	72.52	36.30	63.70	35.92	64.08	17.75	82.25	30.08	69.92	29.57	70.43	22.02	77.98
BLVU	108	M	8.48	91.52	15.50	84.50	28.75	71.25	21.48	78.52	17.11	82.89	6.88	93.12	11.77	88.23	27.43	72.57	25.07	74.93	42.28	57.72	15.24	84.76	17.49	82.51
BLVU	126	M	67.59	32.41	54.68	45.32	76.74	23.26	52.42	47.58	41.22	58.78	35.53	64.47	39.17	60.83	38.98	61.02	14.83	85.17	25.86	74.14	25.22	74.78	50.52	49.48
TUVU	01	F	63.94	36.06	68.31	31.69	72.83	27.17	66.87	33.13	62.99	37.01	53.60	46.40	51.75	48.25	60.98	39.02	50.03	49.97	64.35	35.65	55.55	44.45	57.30	42.70
TUVU	03	F	65.55	34.45	65.56	34.44	72.23	27.77	66.60	33.40	59.14	40.86	60.03	39.97	60.25	39.75	67.10	32.90	61.38	38.62	65.60	34.40	68.23	31.77	69.82	30.18
TUVU	13	F																	45.41	54.59	49.18	50.82	45.42	54.58		
TUVU	06	M	47.51	52.49	50.40	49.60	64.58	35.42	60.40	39.60	50.09	49.91	52.43	47.57	47.90	52.10	50.87	49.13	44.98	55.02	49.55	50.45	43.43	56.57	50.12	49.88
TUVU	60	M	51.45	48.55	61.01	38.99	75.77	24.23	74.05	25.95	64.23	35.77	50.37	49.63	47.74	52.26	52.57	47.43	53.08	46.92	50.45	49.55	46.17	53.83	39.64	60.36
TUVU	75	M	51.02	48.98	62.86	37.14	68.61	31.39	68.05	31.95	65.44	34.56	61.51	38.49	61.17	38.83	61.23	38.77	55.92	44.08	59.89	40.11	47.04	52.96	45.31	54.69
TUVU	90	M	59.60	40.40	71.52	28.48	73.59	26.41	62.64	37.36	56.57	43.43	44.30	55.70	57.63	42.37	57.57	42.43	41.49	58.51	60.14	39.86	53.79	46.21	58.45	41.55
TUVU	91	M	41.51	58.49	57.64	42.36	69.46	30.54	58.74	41.26	53.83	46.17	48.95	51.05	42.34	57.66	57.92	42.08	50.55	49.45	50.99	49.01	37.95	62.05	42.84	57.16
TUVU	123	M	41.52	58.48	54.86	45.14	62.57	37.43	61.19	38.81	66.46	33.54	59.31	40.69	60.03	39.97	57.47	42.53	41.13	58.87	35.35	64.65	39.09	60.91	40.60	59.40
