# Supplementary material for: Fine-scale assessment of home ranges and activity patterns for resident black vultures (Coragyps atratus) and turkey vultures (Cathartes aura)
Source: PLoS One. 2017 Jul 5;12(7):e0179819. doi: 10.1371/journal.pone.0179819 (PMC5497974; doi:10.1371/journal.pone.0179819)
Supplement: S6 Table — (PDF) [file pone.0179819.s010.pdf]

Table S6. Proportion (%) of seasonal diurnal movement states calculated from GPS locations for 9 black vultures and 9 turkey vultures from September 2013 – August 2015. White cell values report diurnal activity rates within that season over two years (i.e., pooled data over year 1 and year 2). Grey cell values report diurnal activity rates within that season for a single year (i.e., no data were collected within that season in either year 1 or year 2). Black cells are seasons within which no data were collected. Summer: June 9 – October 8; Winter: October 8 – February 7; Breeding: February 8 – June 9; Species: BLVU = Black Vulture (*Coragyps atratus*), TUVU = Turkey Vulture (*Cathartes aura*); ID #: patagial tag identification number; Sex: F = female, M = male; Transit: locations wherein the bird was in flight; Stationary: locations wherein the bird was not in flight (i.e., resting, roosting).

.			Summer		Winter		Breeding	
Species	ID #	Sex	Transit	Stationary	Transit	Stationary	Transit	Stationary
BLVU	22	F	46.30	53.70	47.60	52.40	41.82	58.18
BLVU	47	F	23.26	76.74	43.29	56.71	52.22	47.78
BLVU	57	F	34.76	65.24	26.48	73.52		
BLVU	92	F	42.84	57.16	42.36	57.64	49.53	50.47
BLVU	08	M	63.11	36.89	65.58	34.42	67.23	32.77
BLVU	12	M	14.74	85.26	22.93	77.07	30.95	69.05
BLVU	48	M	20.80	79.20	30.51	69.49	24.41	75.59
BLVU	108	M	21.73	78.27	21.41	78.59	21.69	78.31
BLVU	126	M	26.77	73.23	49.48	50.52	55.65	44.35
TUVU	01	F	52.76	47.24	60.13	39.87	66.75	33.25
TUVU	03	F	62.41	37.59	66.73	33.27	64.57	35.43
TUVU	13	F	45.10	54.90				
TUVU	06	M	45.28	54.72	47.51	52.49	56.04	43.96
TUVU	60	M	44.57	55.43	48.52	51.48	67.61	32.39
TUVU	75	M	54.45	45.55	54.06	45.94	65.99	34.01
TUVU	90	M	45.88	54.12	58.69	41.31	63.28	36.72
TUVU	91	M	47.30	52.70	43.93	56.07	58.62	41.38
TUVU	123	M	59.91	40.09	39.49	60.51	58.17	41.83
